# Screening Method for the Selection of Oleaginous Yeast-Producing Gold Nanoparticles

**DOI:** 10.3390/ijms26157534

**Published:** 2025-08-04

**Authors:** Jesus D. Guerra, Diana Mariscal-Nava, Miguel Avalos-Borja, Georgina Sandoval

**Affiliations:** 1 LIBBA Laboratory, Industrial Biotechnology Unit, Research and Assistance Center in Technology and Design of the State of Jalisco, A.C., Av. Normalistas No. 800 Col. Colimas de la Normal, C.P., Guadalajara 44270, Jalisco, Mexico; 2 Advanced Materials Division, Potosin Institute of Scientific and Technological Research, Camino a la Presa de San José 2055, Lomas 4ta Secc, San Luis Potosí 78216, Mexico

**Keywords:** gold nanoparticles, lipolytic yeast, screening, UV–Vis spectroscopy, microbial nanotechnology, biosynthesis

## Abstract

The demand for eco-friendly nanomaterial synthesis has increased interest in biological approaches. Yeast-mediated biosynthesis of gold nanoparticles (AuNPs) offers a sustainable alternative with potential biotechnological applications. This study developed a rapid screening method to identify oleaginous yeast strains able to synthesize AuNPs. A collection of 114 oleaginous yeasts from the LIBBA laboratory was screened. UV–Vis spectroscopy at 530–560 nm was used to assess nanoparticle formation, identifying 20 strains that effectively synthesize AuNP. Electron microscopy confirmed the presence of intracellular and extracellular nanoparticles, with variations in size and morphology. This screening and optimization approach effectively identified promising yeast candidates and refined biosynthesis conditions, providing a foundation for industrial-scale nanoparticle production.

## 1. Introduction

In recent years, nanomaterials have generated great impacts in various areas, such as biomedicine, the agricultural industry, biological sciences, and electronics, due to their unique properties, such as their high surface area and great dispersion in solution, compared with those of bulk materials [1,2]. Among nanoparticles, gold nanoparticles (AuNPs) have interesting properties, such as high stability, low toxicity, biocompatibility, and optical properties [3,4,5]. This makes them interesting for various applications, including drug administration, anticancer and antimicrobial activity, cancer therapy, and bioimaging [5,6,7]. Until a few years ago, AuNPs were synthesized only through physical and chemical methods, which require high temperatures or toxic reagents, limiting their synthesis on a large scale [8,9,10,11]. Currently, the “green” synthesis of gold nanoparticles with the use of biological organisms, such as plants, bacteria, fungi, and yeasts, is preferred as a sustainable alternative [12,13].

Yeasts have been shown to be efficient in the synthesis of AuNPs because of their large amount of protein and reducing enzymes involved in the reduction of metal ions [14,15]. Several species of yeasts, including *Schizosaccharomyces cerevisiae*, *Schizosaccharomyces pombe*, *Pichia jadinii*, *Candida glabrata*, and *Torulopsis* sp., have been identified as producers of AuNPs [3,9,14]. Some oleaginous yeasts such as *Yarrowia lipolytica* have also been reported to synthesize AuNPs [16]. Indeed, the metabolic versatility of oleaginous yeasts allows them to produce a variety of alternative biomaterials in future biorefineries [17,18]

The synthesis of AuNPs by yeast is influenced by factors such as the type of microorganism used, its physiological phase, the pH of the reaction medium, the incubation temperature, the reaction time, and the concentration of the metallic precursor, each of which directly impacts size, morphology, and production efficiency of the resulting nanoparticles [14,19].

Despite advances in biosynthesis of AuNPs, the rapid identification of yeasts that are able to synthesize nanoparticles remains a challenge. Currently, many studies have focused on the characterization of a few species without a standardized screening method. The lack of a systematic protocol to evaluate the production of AuNPs among multiple strains limits the advancement of microbial nanotechnology. Therefore, the development of efficient and reproducible screening methods that allow rapid and efficient identification of strains with high AuNP biosynthetic potential is crucial. This will facilitate their application in biotechnology and nanotechnology, optimizing sustainable large-scale production.

In addition to the identification of nanoparticle-producing strains, various studies have shown that the efficiency and properties of biosynthesized nanoparticles largely depend on physicochemical parameters such as pH, temperature, reaction time, agitation, the physiological phase of the organism, and the concentration of the metal precursor [20,21,22]. These factors can directly influence the nucleation kinetics, size, shape, and stability of the resulting nanoparticles. Therefore, optimizing these conditions is also essential for efficient AuNP production by yeasts.

## 2. Results

### 2.1. Yeast Screening for Gold Nanoparticle Biosynthesis

**Figure 1:**
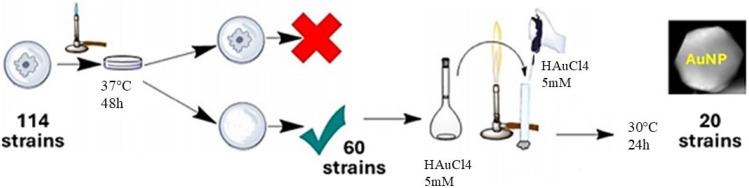
Preselection and screening of yeasts.

A total of 114 yeasts isolated from various biotopes were previously screened for their capacity to accumulate bio-oils [23]. This collection of oleaginous yeasts of unknown genera was subjected to culture at 37 °C, and those able to grow at this temperature were discarded as a biosafety measure, despite their recognized potential for biotechnological application in nanoparticle production. The remaining 60 strains were grown, and their biomass was reacted with gold salt (Figure 1).

**Figure 2:**
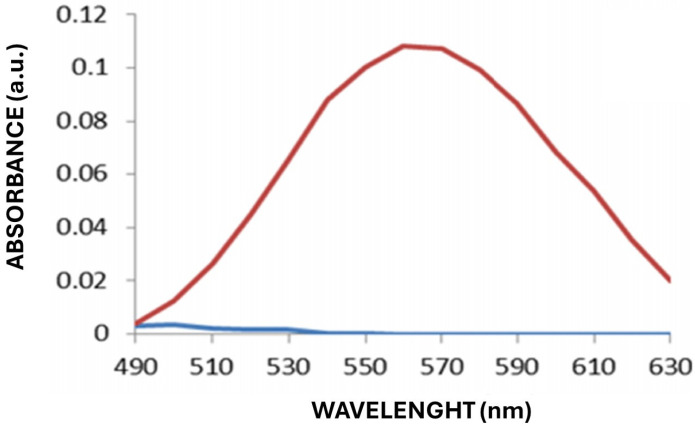
The absorbance spectra corresponding to the positive control, commercial gold nanoparticles (Sigma-Aldrich) suspended in sodium citrate are shown in red and the control (time zero) is shown in blue.

The spectrophotometric results obtained via UV–Vis revealed that, of the 60 strains screened, 20 could produce AuNPs. They presented a change in the UV–Vis signal of the supernatant, showing a peak between 530 and 560 nm, which indicates the formation of AuNPs. Figure 2 shows the absorbance spectrum of the positive control with commercial gold nanoparticles, which was used as a reference to validate the UV–Vis technique and to compare the characteristic plasmon resonance peak of the biosynthesized AuNPs.

The differences in the wavelength of maximal absorbance can be attributed to variations in the reducing activity of each strain and its ability to secrete metabolites involved in the nucleation and growth of the nanoparticles. These results suggest that the selected yeasts have high potential for the biosynthesis of AuNPs, with variability in the efficiency of the process and the characteristics of the resulting nanoparticles. Spectrophotometric data indicated that yeasts with stronger absorption peaks could be associated with more extracellular nanoparticles than those with lower absorbances.

**Figure 3:**
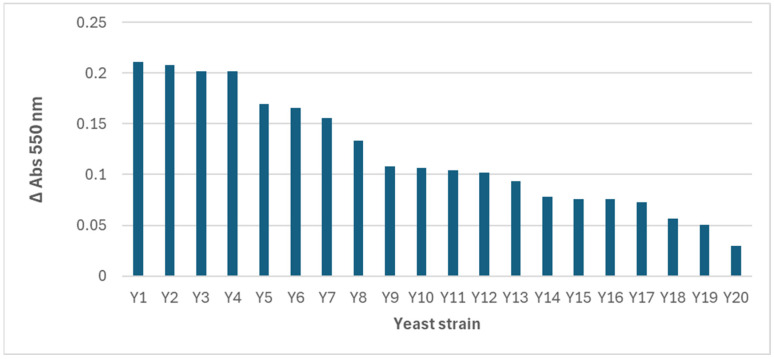
Δ Abs at 550 nm after the reaction of gold salt and yeast biomass.

The spectra obtained revealed differences in the wavelength of the maximum absorbance peak and in the intensity of the signal, which suggests variations in the quantity, size, and morphology of the AuNPs synthesized by each strain. However, as most of the reactions with yeast biomass presented the maximum absorbance at 550 nm, this wavelength was selected, and an absorbance (arbitrary) unit of 0.15 was used as the cutoff, with only 7 of the 20 positive strains surpassing it (Figure 3).

Identification assays of the selected yeasts revealed the following species: *Candida famata*, *Hanseniaspora uvarum*, *Yarrowia lipolytica* (2), *Candida utilis*, *Debaryomyces hansenii*, and *Pichia kudriavzevii*.

**Figure 4:**
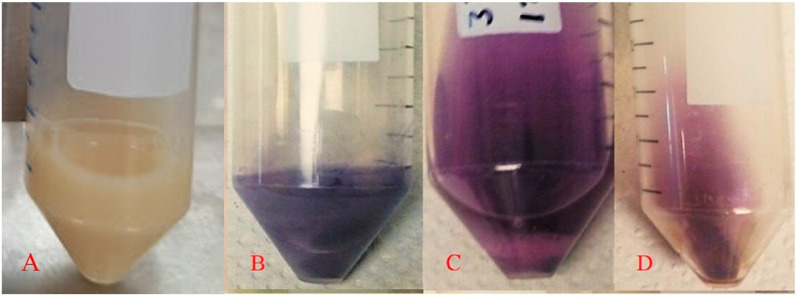
Biomass–gold salt reaction medium. At the beginning (t0) of the reaction (**A**); strains at the end of the reaction: *Yarrowia lipolytica* (**B**), *Candida utilis* (**C**), *Pichia kudriavzevii* (**D**).

Figure 4 shows examples of the biomass–gold salt reaction medium at the beginning (Figure 4A) and at the end of the process for the yeasts *Yarrowia lipolytica* (Figure 4B), *Candida utilis* (Figure 4C), and *Pichia kudriavzevii* (Figure 4D) as representative species, since they were included in all experimental stages presented in this study. Additionally, they exhibited distinct color changes in the reaction medium after biosynthesis, suggesting differences in the morphology and size of the synthesized AuNPs. The biomass–gold salt interaction is directly affected by the strain with which it reacts; therefore, it is a factor that directly affects the formation of AuNPs, resulting in different sizes and shapes, reflected in the different colors of the final AuNP solution. The AuNP solution color was similar to that of the other species (lilac), and a priori they would have the same size and shape. The strains presented an absorption peak at 550–560 nm, associated with an intense purple–blue coloration, indicating similarities in the optical properties of the AuNPs produced.

### 2.2. Thin-Section Microscopy

**Figure 5:**
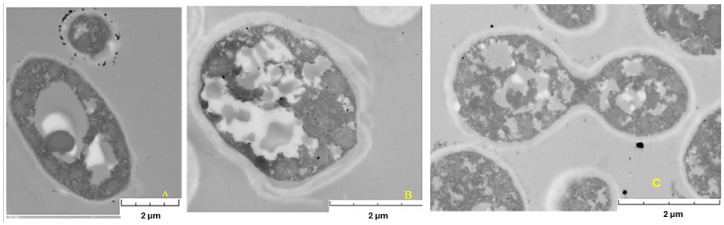
Micrographs observed by TEM with the thin-section technique for the yeasts *Yarrowia lipolytica* (**A**), *Pichia kudriavzevii* (**B**), and *Candida utilis* (**C**).

The thin-section technique [24] was used to study the location of the AuNPs by transmission electron microscopy (TEM) [15]. Micrographs of the yeast biomass after the reaction with gold salt (Figure 5) confirmed that AuNPs were mostly on the cell surface and in the supernatant, suggesting a mechanism of extracellular biosynthesis. For example, in the yeast *Yarrowia lipolytica* (Figure 5A), a uniform distribution of AuNPs was observed around the cell membrane, with variations in size and shape, in addition to the presence of large vacuoles, which could indicate a response to stress induced by the golden salt.

Micrographs also revealed differences in cell morphology between the strains analyzed. *Pichia kudriavzevii* (Figure 5B) presented a plasmatic thick membrane. However, the integrity of the cell seems to have been affected, since, in addition to presenting a thick membrane, it presented deformities and vacuoles of different sizes. Although a slightly thick membrane was also observed in *Candida utilis* (Figure 5C), it could be considered the least affected, since it did not present large vacuoles or deformities in its structure and even buds, indicating cell viability.

On the basis of the micrographs, cellular stress conditions are generated when the biomass interacts with the gold salt, which varies with each strain. Therefore, the stress conditions generated during the biomass–substrate interaction and involved in the generation of gold nanoparticles should be considered in the biosynthesis of AuNPs by yeasts.

### 2.3. Transmission Electron Microscopy (TEM)

**Figure 6:**
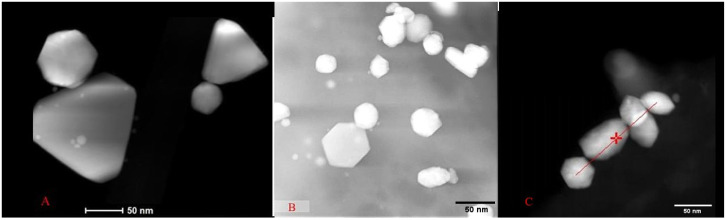
TEM micrographs of the AuNPs synthesized by the yeasts *Yarrowia lipolytica* (**A**), scale bar: 50 nm); *Pichia kudriavzevii* (**B**), scale bar: 50 nm; and *Candida utilis* (**C**), scale bar: 50 nm.

TEM micrographs revealed similarities and variations in the sizes and shapes of the AuNPs synthesized by each strain. For the yeast *Y. lipolytica*, nanoparticles ranging from 40 to 120 nm in diameter with hexagonal and triangular shapes were observed (Figure 6A). For the AuNPs synthesized by the yeast *P. kudriavzevii* (Figure 6B), the diameters were between 30 and 140 nm, presenting hexagonal shapes, and quasispheroidal structures. *C. utilis*-synthesized AuNPs (Figure 6C) were smaller. Indeed, nanoparticles with sizes between 15 and 50 nm were identified, along with agglomerates up to 70 nm in size, exhibiting hexagonal and rounded shapes.

### 2.4. Energy Dispersive Spectroscopy (EDS) Analysis

**Figure 7:**
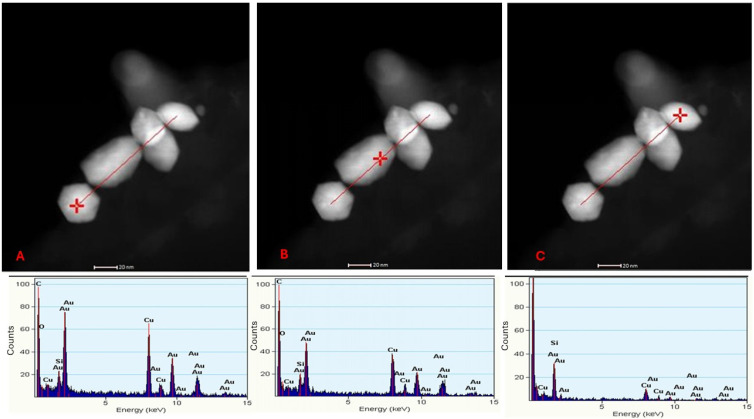
TEM micrograph of the AuNPs biosynthesized by the yeast *C. utilis*, where a point along the trace was analyzed at different locations (**A**–**C**), with its EDS spectrum corresponding to each point. Scale bar: 20 nm.

To confirm the elemental chemical composition of the observed nanoparticles, EDS was performed on the synthesis by the yeast *C. utilis* at three specific points along a linear trace marked in the micrographs (Figure 7). The points analyzed, indicated with a red cross, corresponded to the EDS spectra presented in Figure 7A–C. In each spectrum, characteristic peaks of gold [25] were identified, confirming the chemical nature of the observed nanoparticles.

In addition to the gold peaks, copper (Cu) and silicon (Si) signals were detected at the three points analyzed [17]. These signals are attributed to the support grid used for sample preparation, a common phenomenon in EDS analysis that is not related to AuNPs or the biological matrix of yeasts. This finding underscores the importance of considering experimental artifacts when interpreting elemental composition results.

### 2.5. Scanning Electron Microscopy (SEM)

**Figure 8:**
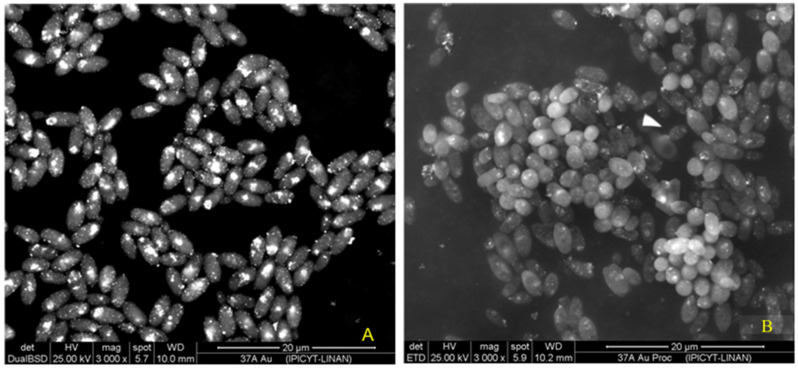
SEM micrographs of *Candida utilis* cells after reaction with HAuCl_4_. Observed with BSD (**A**) and SED (**B**).

SEM observations of *C. utilis* biomass (Figure 8) revealed the presence of gold nanoparticles (AuNPs) on the cell surface. By means of a backscattered electron detector (BSD), the atomic composition of the elements present in the sample was differentiated, showing a signal in the regions where gold atoms predominated, which resulted in bright spots on the surface of the cells (Figure 8A). This detector confirmed the presence of AuNPs in the samples. In addition, the cells were in a good physiological state (without cell lysis), and some were in a budding state.

On the other hand, the secondary electron detector (SED) showed the surface topography of the cells (Figure 8B). Although the AuNPs were also visible, the signal was less intense in the organic matter regions due to their low conductivity. Since the samples were not coated during processing, the surfaces occupied by organic matter gave a less intense signal than did those covered by gold, limiting the resolution of the surface characteristics of the cells. However, the high acceleration voltage used allowed the detection of secondary electrons from the surface layers, confirming the presence of AuNPs on the cell surface. In addition, with this image, we can see that the cells are alive, cell lysis is not observed, and budding can even be observed. These findings suggest that the screened *Candida utilis* strain is very resistant to gold salt. Therefore, this strain was selected for further exploration of parameters for biosynthesis.

### 2.6. Effect of Reaction Conditions on the Biosynthesis of AuNP

To evaluate the effects of different physicochemical conditions on the biosynthesis of gold nanoparticles (AuNPs), four experimental reactions were compared using the *C. utilis* strain as a model. The production of AuNPs was measured via UV–Vis spectroscopy which is an indicator of the maximum absorbance at 530 nm, which is characteristic of the presence of AuNPs.

**Figure 9:**
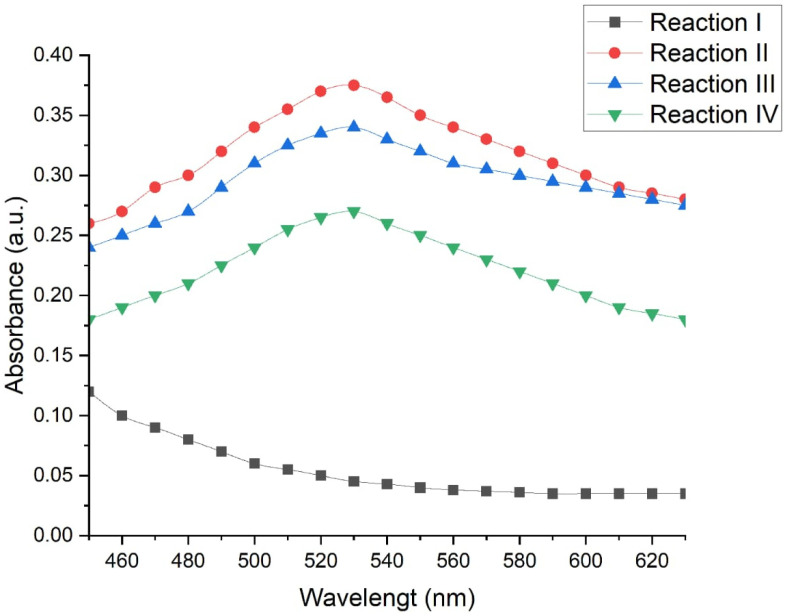
Absorption spectra of reactions I, II, III and IV in the formation of AuNP by the yeast *C. utilis*.

Figure 9 shows the absorbance spectra obtained under each of the evaluated conditions. A defined peak was observed at 530 nm in reactions II, III, and IV, indicating the formation of AuNPs. On the other hand, reaction I did not present such a peak, which suggests a low or no synthesis of nanoparticles under these conditions.

Reaction II (50 °C, 130 rpm, 48 h) had the highest absorbance, with a value of 0.350 absorbance units (a.u.), which indicates a more efficient synthesis of nanoparticles under conditions of moderate temperature, constant agitation, and prolonged incubation time. Reaction III (50 °C, without agitation, 24 h) presented a slightly lower absorbance (0.325 a.u.), which suggests that the reduction in the reaction time and the absence of agitation slightly decreased the efficiency of the process. Reaction IV (70 °C, without agitation, 6 h) resulted in the lowest absorbance of the productive conditions (0.235 a.u.), which indicates that excessive temperatures, together with short times without agitation, negatively affect the synthesis of AuNP.

Statistical analysis of the spectrophotometric data confirmed that the evaluated reaction conditions significantly influence the production of gold nanoparticles. A multiple comparison test was applied via Fisher’s LSD with a confidence level of 95%, which allowed the identification of statistically significant differences between the evaluated methods (Table 1).

Reaction II presented significantly higher absorbance values than the other conditions did (*p* < 0.05), confirming its superior efficiency for the biosynthesis of AuNPs. In contrast, reaction I was significantly different (and lower) with respect to reactions II, III, and IV, which supports the absence of the characteristic peak at 530 nm.

Likewise, significant differences were detected between reactions II and IV, with a difference value of 0.0998 ± 0.0796. No statistically significant differences were observed between reactions II and III or between reactions III and IV, which suggests that the reduction in the incubation time and the absence of agitation have a moderate effect but are not always critical and that the results depend on other factors such as temperature.

## 3. Discussion

According to the results, of the 60 strains evaluated, 20 presented an absorption peak between 530 and 560 nm, which, according to Zhao et al. [15] and Xu et al. [4], is indicative of the presence of AuNPs. Owing to the implementation of the UV–Vis spectrophotometric method, it was possible to identify yeasts capable of producing AuNPs in a fast, viable, and reproducible way, thus validating the proposed screening method.

SEM analysis confirmed the presence and extracellular synthesis of the nanoparticles. This finding agrees with previous studies [14], where the extracellular synthesis of AuNPs by reducing metabolites of the yeast *Yarrowia lipolytica* NCIM 3589 was mentioned.

The size of the AuNPs observed via transmission electron microscopy ranged from 20 to 150 nm in diameter, with various morphologies, such as rods, hexagonal, triangular, and round. This observed variability suggests that the AuNPs produced may be influenced by specific physiological factors of each strain such that there are different cellular mechanisms involved in the reduction of HAuCl_4_ and in the nucleation of the particles [26,27]. In addition, the colors observed in the supernatant and the maximum absorption wavelengths are decisive and are linked to the shapes and sizes of the NPs, which serves as a first indication of the production of AuNPs.

On the basis of the TEM micrographs obtained via the thin-section technique, the interaction between the biomass and the gold salt generated cellular stress, which affected each strain differently through the formation of enlarged vacuoles or cellular deformations and influencing their ability to produce AuNPs [26]. Therefore, the reaction with gold salt seems to be an important factor, since cellular stress acts as a mechanism in the synthesis of AuNPs. Given that different responses are observed between strains, it is possible that the magnitude of stress and its impact vary, which could explain the differences in the production of AuNPs. This variability prompted us to explore how the cellular stress induced by the interaction with gold salt might be involved in the efficiency of AuNP biosynthesis, opening the door to future research on this process [22]. Sathiyaraj et al. [28] used the EDS technique to identify the signals corresponding to gold. In this research EDS analysis confirmed the chemical nature of the nanoparticles as gold, ruling out interference by materials from the medium or the cell. This analysis reinforces the reliability of screening to detect true biologically synthesized AuNPs.

In addition to the screening process, the biosynthesis parameters of the AuNPs were optimized for the *Candida utilis* strain, with the aim of evaluating the influences of agitation, temperature, and reaction time on the production of AuNPs. Previous studies have shown that physicochemical variables such as pH, temperature, and biomass concentration significantly affect the synthesis of metallic nanoparticles. Balakumaran et al. [21] reported that these conditions directly impact the production, size, and shape of nanoparticles. In the present study, the results showed that reactions II and III, both carried out at 50 °C, presented the highest absorbances, which indicates a relatively high concentration of AuNPs. However, the difference between the two methods was the presence of agitation (130 rpm in reaction II and without agitation for reaction III), which allows us to infer that the combination of temperature and adequate agitation promotes greater production when the mixture is in contact with the metallic precursor together with the biomass. These findings coincide with what was reported by Pimprikar et al. [22], who reported that the concentration of biomass and the metallic precursor also influence the shape and size of the AuNPs generated. In addition, Krishnaraj et al. [20] showed that adjusting the pH and concentration of the metallic salt is key to achieving rapid and controlled synthesis, obtaining stable nanoparticles with sizes smaller than 50 nm in just 30 min at a pH of 7. While adjusting parameters such as pH and gold salt concentration is known to be critical for achieving rapid and controlled synthesis, these factors were kept constant in this study, as our previous exploration of these factors led to the choice of pH 3.5 and 5 mM gold salt. Therefore, the observed differences in nanoparticle size, stability, morphology, quantity, and speed of formation can be directly attributed to the variables evaluated: temperature, time, and agitation. This highlights the importance of properly adjusting these conditions to control the size, stability, morphology, yield, and rate of nanoparticle formation. These findings highlight the importance of maintaining continuous contact between the biomass and the gold ions, as well as allowing the time necessary for their reduction and growth. In contrast, high temperatures, short reaction times, and the absence of agitation markedly decreased the production of AuNPs, which suggests that excess stress or limited reaction kinetics can negatively affect the biosynthetic process. These results reinforce the need to optimize not only the biological system but also the physicochemical conditions of the environment to achieve reproducible and scalable synthesis of nanoparticles.

Although the chemical synthesis of metallic nanoparticles can be easy, additives such as polyvinylpyrrolidone are usually needed to stabilize them [29]. Biological synthesis, cells can produce natural stabilizers (enzymes, phenols, sugars, etc.) that can participate in both reduction and stabilization [30].

On the other hand, oleaginous yeasts have been reported to grow rapidly from low-cost and industrial waste media, such as molasses, crude glycerol, or agro-industrial waste. They also tolerate a wide range of environmental conditions, which facilitates their cultivation in batch bioreactors or in continuous systems without the need for strict controls, which makes them feasible for scale-up processes [23,31]. In addition, lipid accumulation in oleaginous yeast could protect them from nanoparticles. Moreover, some nanoparticles even enhance lipid productivity in yeasts [32].

## 4. Materials and Methods

### 4.1. Materials, Microorganisms, and Culture Conditions

Hydrogen (III) tetrachloroauric acid (HAuCl_4_·3H_2_O) and gold nanoparticles (AuNPs) were obtained from Sigma-Aldrich^®^ (Merck, Naucalpan de Juárez, Mexico).

The oleaginous yeasts used in this study were selected from the collection of strains of the LIBBA lab, Center for Research and Assistance in Technology and Design of the State of Jalisco, AC (CIATEJ), and were initially incubated in YPD media (yeast extract, peptone, dextrose) under the conditions described in the Results Section (depending on the screening or exploring reaction conditions stage).

### 4.2. Yeast Identification

Internal transcribed spacer 1 (ITS1) was chosen to determine the identity of the screened yeasts, using the methodology described elsewhere [25]. Yeast isolates were amplified via colony PCR using universal primers [33], and a sequence of PCR products were compared via BLAST version 1.1 on available databases [34].

### 4.3. Yeast Screening in Gold Nanoparticle Biosynthesis

A total of 114 initial strains were selected, of which 54 were excluded because of their growth capacity at 37 °C, to rule out possible pathogenic cells in humans. Consequently, the experiment continued with 60 strains.

The 60 strains were cultured in the YPD liquid medium (pH 4.5) in 250 mL flasks, with orbital shaking at 130 rpm at 30 °C for 24 h. The biomass obtained was separated by centrifugation and washed three times with sterile water.

To evaluate the production of AuNPs, the biomass obtained was put in contact with a chloroauric acid solution (HAuCl_4_) at a concentration of 5 mM at 30 °C, with orbital shaking (130 rpm) at a pH of 3.0 for 24–48 h. As experimental controls, samples of biomass without HAuCl_4_ and HAuCl_4_ without biomass were included.

### 4.4. Characterization of the Nanoparticles

#### 4.4.1. UV–Vis Spectrophotometry

The production of the gold nanoparticles was detected via UV–Vis spectrophotometry using a SpectraMax i3x microplate reader (Molecular Devices, San Jose, CA, USA). Spectrophotometric scans were performed in the range of 430–630 nm at 10 nm intervals, and the readings were compared with those obtained with a commercial standard of Sigma-Aldrich^®^ (Merck, Mexico) gold nanoparticles suspended in sodium citrate and with the gold salt without reduction. The presence of a characteristic absorption peak in the range of 520–560 nm was considered a criterion for the production of AuNPs, which indicates the production of stable AuNPs in the solution [35].

#### 4.4.2. TEM

Samples with a volume of 1 mL were sonicated for 5 min at 25 °C. Subsequently, the supernatant was centrifuged at 10,000 rpm for 10 min to remove impurities. The pellet was then resuspended in 1 mL of distilled water and sonicated again for 15 min at 25 °C.

For the preparation of the samples, 5 µL of the suspension was deposited on a lacey carbon grid, left to dry for 12 h inside a Petri dish covered with aluminum foil, and then irradiated with a 12 W incandescent lamp. In addition, samples of cells used for the production of AuNPs were prepared for analysis via the thin-section technique (ultramicrotomy) to evaluate the intracellular and extracellular locations of the nanoparticles.

The samples were analyzed via a high-resolution transmission electron microscope (HR-TEM FEI Tecnai F30, Thermo Fisher Scientific, Waltham, MA, USA) operating at 300 kV at the LINAN-IPICYT facility. Image acquisition and analysis were performed via Gatan DigitalMicrograph^®^ and ImageJ version 1.54 (NIH, Bethesda, MD, USA).

#### 4.4.3. SEM

For the morphological observation of the nanoparticles and their interaction with the cells, the samples were processed by diluting them according to the amount of biomass present. One to three washes were performed using phosphate-buffered saline (PBS), depending on the cell concentration, and the biomass in each wash was resuspended and centrifuged for 10 min. The samples were then fixed with 2.5% glutaraldehyde for 1 h at 4 °C and subsequently dehydrated through a graded ethanol (70%) for 10 min at each step. These treatments effectively removed residual unreduced gold salt. Finally, 30 µL of the sample was deposited at three different points (10 µL at each point) on a carbon strip mounted on an aluminum pin. The samples were allowed to dry for 12 h inside a box closed with aluminum foil and irradiated with a 12 W incandescent lamp before analysis via a scanning electron microscope. SEM analysis was performed using an FEI-ESEM QUANTA FEG microscope at LI-NAN-IPICYT. Images were processed and analyzed with ImageJ (NIH, USA).

#### 4.4.4. Effect of Reaction Conditions on AuNP Biosynthesis

A preliminary evaluation was conducted to identify the physicochemical conditions that favor the biosynthesis of the AuNPs, considering temperature, time, and agitation as influencing factors. The biosynthesis conditions were carried out using the strain LGS01.1, previously identified as a producer of AuNPs, as a model. The response variable was the UV–Vis absorbance (530 nm) corresponding to the formation of AuNPs.

The pH of the reaction medium was controlled at 3.5, and the cell concentration was 1 × 10^9^ cells/mL. The experimental parameters evaluated were agitation, reaction time, and reaction temperature, the values of which are described in Table 2.

The results obtained allowed the identification of the appropriate conditions to maximize the spectrophotometric signal associated with the production of AuNPs. Statistical analysis was conducted via Statgraphics Centurion XVI.I software.

## 5. Conclusions

Rapid identification via UV–Vis spectroscopy of AuNPs biosynthesized by yeasts represents a significant advance in the systematic identification of microorganisms capable of producing NPs, especially when the production capacity of various strains is tested. In this study, 20 out of the 60 strains analyzed presented positive absorbance peaks between 530 and 560 nm, allowing efficient screening and reducing the identification time of positive strains, opening new opportunities in the design of eco-friendly biotechnological processes. On the other hand, the gold nanoparticles synthesized by yeast have different spectrophotometric absorption lengths for each strain, which suggests that the AuNPs have different shapes and/or sizes. The differences observed in the cellular responses suggest that the gold salt, the type of strain, and its physiological state could be related to the morphology of the AuNPs produced.

Moreover, the optimization of biosynthesis parameters allowed the identification of key experimental conditions to maximize the efficiency of the AuNP production by the screened yeast *Candida utilis*. Specifically, a temperature of 50 °C, agitation at 130 rpm, and a reaction time of 48 h resulted in the highest spectrophotometric signal, suggesting greater efficiency in nanoparticle formation under these conditions. These findings demonstrate that fine adjustments in variables such as agitation, reaction time, and temperature influence not only the quantity of nanoparticles produced but also their morphology and size. The protocol described in this work uses live biomass without requiring cell disruption or chemical additives, which makes the process simpler and less costly. Therefore, this process could be adapted to larger scale applications. Future work could focus on exploring the influence of each physicochemical variable through factorial design approaches and studies of the structure–function relationships of AuNPs.

## Figures and Tables

**Table 1:** Multiple comparisons between reaction conditions were performed via Fisher’s LSD test (α = 0.05). * indicates statistically significant difference; ns = not significant.

Comparison	Difference	±Limit	Significance
Reaction I–II	−0.2918	0.0796	*
Reaction I–III	−0.2632	0.0796	*
Reaction I–IV	−0.1929	0.0796	*
Reaction II–III	0.0286	0.0796	ns
Reaction II–IV	0.0998	0.0796	*
Reaction III–IV	0.0712	0.0796	ns

**Table 2:** Experimental conditions for physicochemical optimization.

Factors	Reaction I	Reaction II	Reaction III	Reaction IV
pH	3.5	3.5	3.5	3.5
Agitation	130 rpm	130 rpm	0 rpm	0 rpm
Time	48 h	48 h	24 h	6 h
Temperature	30 °C	50 °C	50 °C	70 °C

## Data Availability

The original contributions presented in this study are included in the article. Further inquiries can be directed to the corresponding author.

## Abbreviations

AuNP	Gold nanoparticles
SEM	Scanning electron microscopy
EDS	Energy dispersive spectroscopy
TEM	Transmission electron microscopy
BDS	Backscattered electron detector
SED	Secondary electron detector
Δ Abs	Absorbance difference with respect to the control
